# Intravenous pretreatment with emulsified isoflurane preconditioning protects kidneys against ischemia/reperfusion injury in rats

**DOI:** 10.1186/1471-2253-14-28

**Published:** 2014-04-16

**Authors:** Zhaojun Qin, En Lv, Leyun Zhan, Xiangfei Xing, Jianli Jiang, Min Zhang

**Affiliations:** 1 Department of Anesthesiology, Three Gorges University People’s Hospital, Yichang, China, No. 4, Hudi Street, Xiling District, Yichang 443000, Hubei, People’s Republic of China

**Keywords:** Emulsified isoflurane, Acute renal ischemia, Preconditioning, Inflammation, Oxidative stress

## Abstract

**Background:**

Emulsified isoflurane (EIso) is a novel intravenous general anesthetic, which can provide rapid anesthetic induction and recovery. EIso preconditioning could attenuate heart, lung and liver ischemia/reperfusion (I/R) injury. We tested the hypothesis that intravenous pretreatment with EIso would protect kidneys against I/R injury by inhibiting systemic inflammatory responses and improving renal antioxidative ability.

**Methods:**

Rats were randomly divided into these six groups: sham, I/R, intralipid, 1, 2 or 4 ml/kg EIso. Rats were subjected to 45 min left renal pedicle occlusion followed by 3 h reperfusion after right nephrectomy. Rat were treated with intravenous 8% EIso with 1, 2 or 4 ml/kg, or 30% intralipid with 2 ml/kg for 30 min before ischemia, respectively. After reperfusion, renal functional parameters, serum mediator concentrations and markers of oxidative stress in kidney tissues were determined, and renal histopathological analysis were performed.

**Results:**

Serum creatinine, blood urea nitrogen, cystatin c, tumor necrosis factor-α, interleukin-6, and interleukin-10 concentrations were significantly increased after renal I/R as compared to the sham group. So was renal tissue MDA content and histological scores, but renal tissue SOD activity was decreased. Additionally, severe morphological damages were observed in these study groups. In contrast, 2 or 4 ml/kg EIso reduced serum creatinine, blood urea nitrogen, cystatin c, tumor necrosis factor-α, and interleukin-6 levels, decreased renal tissue MDA content and histological scores, increased serum interleukin-10 level and tissue SOD activity as compared to the I/R, intralipid and 1 ml/kg EIso groups. Renal morphological damages were alleviated after pretreatment of 2 or 4 ml/kg EIso.

**Conclusions:**

Intravenous EIso produces preconditioning against renal I/R injury in rats, which might be mediated by attenuating inflammation and increasing antioxidation ability.

## Background

Ischemia/reperfusion (I/R) injury is an important cause of intrinsic acute kidney injury in both allograft and native kidney [1], which accounts for high mortality and morbidity in patients [2]. Improving the ability of the kidney to tolerate I/R injury would have important implications. An increasing number of drugs, including inhalational anesthetics, have a pharmacological preconditioning effect in organs [3-8] as ischemic preconditioning. Inhaled isoflurane was showed to reduce acute renal injury induced by I/R, and the mechanisms involved anti-inflammation [9], the inhibition of protein kinases [10], the activation of adenosine triphosphate-dependent potassium channels [9], the release of renal tubular transforming growth factor-β 1 [7], ecto-5′-nucleotidase (CD73) and adenosine [11], and/or the upregulation of hypoxia inducible factor-1 α [12]. The overall mechanism of organ protection by volatile anesthetics is likely to be multifactorial [13,14], and it has been the focus of intense investigation.

Emulsified isoflurane (EIso) is a novel intravenous general anesthetic, which has been the subject of recent research because it was found to eliminate the need for specific ventilatory circuits, provide rapid anesthetic induction and recovery, have remarkable hemodynamic stability [15] and low environmental pollution and tissue toxicity [16]. It can be administered intravenously rather than as an inhalant. It is easier to control the depth of anesthesia than isoflurane inhalation. Rao et al. [17] demonstrated that 8% EIso had a myocardial protective effect on I/R injury similar to that of inhaled isoflurane in rabbits. It is also showed that intravenous pretreatment with 2 or 4 ml/kg EIso affords effective protection against myocardial ischemia in rats by inhibiting apoptosis [15]. Lv et al. [18] demonstrated that EIso reduced lung injury induced by hepatic I/R by decreasing tumor necrosis factor-α (TNF-α) level and down-regulation of intercellular adhesion molecule-1 in the lung tissue. Recently, it was reported that EIso postconditioning could produce cardioprotection against myocardial I/R injury in rats by the inhibition of apoptosis [19] and the preservation of mitochondrial function [20]. Whether EIso administered before ischemia could protect kidneys against reperfusion injury remains unknown.

Therefore, we have hypothesized that EIso could protect the kidney against I/R injury by inhibiting systemic inflammatory responses and improving renal antioxidative ability. In this study we set out to examine the effects of different preconditioning doses on renal I/R injury, and systemic mediators release and oxidative stress reaction in the model of renal I/R injury in rats. We measured renal functional parameters, serum mediators involved in inflammation, markers of oxidative stress in kidney tissues and outcome of acute renal I/R injury.

## Methods

### Animal preparation

Animals were treated according to the Guidelines of Animal Experiments from the Committee of Medical Ethics, National Health Department of China and with approval of the Institutional Animal Care and Use Committee of Wuhan University. Adult male Sprague Dawley rats weighing 220–300 g were obtained from the Animal Experiment Centre of Wuhan University. Rats were acclimated for 1 week before experiments, with unrestricted access to water and food.

Following a 12 h fasting period, rats were anesthetized with 1% sodium pentobarbital (Sigma Chemical Company, St. Louis, MO, USA) 5 ml/kg given intraperitoneally. Anesthesia was maintained by intravenous 1% sodium pentobarbital 1–2 ml/kg. During surgical procedures, rats were supine on a heating pad and body temperature was maintained at 36–38°C. A 24 gauge venous catheter was inserted into the right jugular vein, and Ringer’s lactate solution was administered at a bolus dose of a 0.5 ml and continued as an infusion at 8 ml/kg/h. Acute renal ischemia was established as described previously [9]. Briefly, after a midline laparotomy and right nephrectomy, left renal ischemia was induced by unilateral renal pedicle clamping for 45 min with an atraumatic microaneurysm clamp. After 45 min of left renal ischemia, the occlusion clip was removed and renal reperfusion was allowed. Successful renal ischemia is defined as the color of kidney is changed from bright red to dark red; on the contrary, successful renal reperfusion is defined as the color is changed from dark red to bright red.

### Preparation of EIso

EIso (8% vol./vol.) was manufactured by Yichang Humanwell Pharmaceutical Co. Ltd (Yichang, China) according to the procedures described previously [21], which was kindly donated by Prof. Jin Liu from the Laboratory of Anesthesiology and Critical Care Medicine, West China Hospital, Sichuan University (Chengdu, China). Briefly, 1.6 ml liquid isoflurane (Abbott Laboratories, Kent, UK) and 18.4 ml 30% intralipid (fat emulsion injection, Huarui Pharmaceutical Co., Ltd. Wuxi, China) was mixed in a 20 ml glass ampoule and sealed using an alcohol blowtorch. The ampoule was then vigorously shaken on a vibrator for 15 min to solubilize isoflurane into a lipid emulsion. The EIso ampoule was stored at 4°C until use. The ampoule were opened and warmed to 37°C for 2 h before intravenous administration. And the residual drug was discarded. The stability of EIso was determined by gas chromatography before the experiment (Aligent 4890 D; Tegent Technology Ltd., Shanghai, China).

### Experimental protocol

After stabilization, forty-two rats were randomly allocated to the following six treatment groups: sham, I/R, intralipid, and EIso groups (1, 2 or 4 ml/kg EIso).

Sham group (n = 7), Group S: animals were only underwent right nephrectomy and exposured of the left renal pedicles, but were not subjected to any I/R. No additional medication was given, only a Ringer’s lactate solution infusion was maintained.

I/R group (n = 7), Group I/R: the left renal I/R injury models were induced as described above. No additional medication was given, only a Ringer’s lactate solution infusion was maintained.

Intralipid group (n = 7), Group I: 30% intralipid was intravenously infused at a dose of 2 ml/kg for 30 min before renal ischemia. Infusion was discontinued 15 min prior to the renal pedicle occlusion.

EIso groups (n = 7), Group EIso 1, 2 or 4 ml/kg: EIso (8% vol./vol.) was intravenously administered at a dose of 1, 2 or 4 ml/kg for 30 min prior to the renal ischemia, respectively. Infusion was followed by a 15-min washout period before the renal pedicle occlusion.

Blood samples were taken from the abdominal aorta 3 h after reperfusion. After blood samples were clotted for 2 h at room temperature, the serum was harvested immediately by centrifugation of 1000 g for 30 min at 4°C and stored at -70°C for biochemical analysis. Creatinine (Cr), blood urea nitrogen (BUN) (Ningbo Rui Biotechnology Co., Ltd. Ningbo, China) and cystatin c (CYC) (Shanghai Jingyuan Medical Instruments Co., Ltd. Shanghai, China) were measured in serum with an AU5400 autoanalyzer (Beckman, Tokyo, Japan). TNF-α, interleukin-6 (IL-6) and IL-10 were measured in serum by enzyme linked immunosorbent assay (ELISA) (Elaboratescience Biotechnology Co., Ltd. Wuhan, China), according to the manufacturer guidelines.

At the end of the experiment animals were sacrificed with an overdose sodium pentobarbital. The left kidneys were bisected along the long axis and cut into two equal-sized slices. Tissue sample from one half kidneys was homogenized in 0.1 mol/L phosphate buffer (pH 7.4) at a ratio of 1:10 weight for volume. After centrifugation at 4,000 g for 5 min, the supernatant was extracted for measurement of malondialdehyde (MDA) content and superoxide dismutase (SOD) activity. As markers of oxidative stress, MDA content was determined by the thiobarbituric acid method, whereas SOD activity was evaluated according to the xanthine oxidase method. MDA content and SOD activity in renal tissue were measured with a spectrometer using commercially available kits (Jiancheng Biologic Project Co., Nanjing, China). Renal tissue protein concentration was determined by the bicinchoninic acid method (Beyotime Institute of Biotechnology, Shanghai, China).

Another half kidney was fixed in a 4% neutral paraformaldehyde solution for 24 h. After automated dehydration through a graded alcohol series, transverse kidney slices were embedded in paraffin. Five-micron-thick sections were cut by microtome, stained with hematoxylin-eosin. A quantitative histological assessment under a light microscope (BX50 Olympus; Toyko, Japan) was performed by an experienced pathologist who was blinded to the treatment conditions. A grading scales (scores of 0 to 4) for assessment of necrotic injury of the proximal tubules was used for the histopathological assessment of damage in each animal, as outlined by Jablonski et al. [22] (Table 1). And then a mean score of each group was calculated (scores potentially ranging from 0–4).

**Table 1:** **Numerical scoring system used in the histopathological evaluation of renal ischemia **[22]

**Grade**	**Degree of the proximal convoluted tubule necrosis**
0	Normal histology
1	Mitoses and necrosis of individual cells
2	Necrosis of individual cells in adjacent proximal convoluted tubules, with survival of surrounding tubules
3	Necrosis confined to the distal third of the proximal convoluted tubule with a band of necrosis extending across the inner cortex
4	Necrosis affecting all three segments of the proximal convoluted tubule

The numerical scoring system for the histopathological evaluation of renal ischemia was first outlined by Jablonski et al. [22]. Histological assessment of kidney slices stained with hematoxylin-eosin was performed by a pathologist under a light microscope. Grade 0 indicates normal proximal convoluted tubule, Grade 4 indicates severly damaged proximal convoluted tubule.

### Statistical analysis

Data are presented as mean ± standard deviation. Statistical analyses were performed by one-way analysis of variance followed by the Student-Newman-Keuls test for multiple comparisons. A value of P < 0.05 was considered statistically significant. All analyses were performed using SPSS 18.0 statistical software (SPSS Inc., Chicago, IL, USA).

## Results

### Renal function parameters

Kidneys subjected to 45 min ischemia and 3 h reperfusion significantly increased serum Cr, BUN and CYC concentrations as compared to the sham group (P < 0.05) (Table 2). Both 2 and 4 ml/kg EIso preconditioning markedly decreased serum Cr, BUN and CYC concentrations compared with the I/R, intralipid and 1 ml/kg EIso groups (P < 0.05) (Table 2). There were no significant differences between the 2 and 4 ml/kg EIso groups about these renal function parameters (P > 0.05). Meanwhile, no significant differences were observed in these parameters among the I/R, intralipid and 1 ml/kg EIso groups (P > 0.05).

**Table 2:** Renal functional parameters, markers of inflammation and oxidative stress

	**S**	**I/R**	**I**	**EIso 1 ml/kg**	**EIso 2 ml/kg**	**EIso 4 ml/kg**
Cr (μmol/L)	24 ± 5	98 ± 6^*^	94 ± 15^*^	96 ± 11^*^	61 ± 6^*†‡§^	63 ± 3^*†‡§^
BUN (mmol/L)	6.5 ± 1.6	14.5 ± 4.7^*^	13.9 ± 4.5^*^	14.0 ± 3.5^*^	9.3 ± 2.3^*†‡§^	9.6 ± 4.2^*†‡§^
CYC (mg/L)	1.35 ± 0.16	2.42 ± 0.27^*^	2.5 ± 0.15^*^	2.4 ± 0.24^*^	1.71 ± 0.10^*†‡§^	1.87 ± 0.14^*†‡§^
TNF-α (pg/ml)	47.6 ± 1.0	121.0 ± 6.8^*^	114.9 ± 10.1^*^	120.2 ± 11.2^*^	80.5 ± 5.4^*†‡§^	84.8 ± 7.7^*†‡§^
IL-6 (pg/ml)	36 ± 6	108 ± 9^*^	98 ± 6^*^	96 ± 4^*^	67 ± 6^*†‡§^	71 ± 6^*†‡§^
IL-10 (pg/ml)	19 ± 6	57 ± 12^*^	57 ± 11^*^	67 ± 7^*^	85 ± 10^*†‡§^	83 ± 7^*†‡§^
MDA (nmol/mg)	1.32 ± 0.19	2.05 ± 0.31^*^	1.91 ± 0.35^*^	1.98 ± 0.32^*^	1.61 ± 0.28^*†‡§^	1.62 ± 0.17^*†‡§^
SOD (U/mg)	117 ± 10	78 ± 12^*^	79 ± 13^*^	80 ± 17^*^	97 ± 7^*†‡§^	98 ± 10^*†‡§^

Serum concentrations of creatinine (Cr), blood urea nitrogen (BUN), cystatin c (CYC), tumour necrosis factor-α (TNF-α), interleukin-6 (IL-6), IL-10, and renal tissue malondialdehyde (MDA) content and superoxide dismutase (SOD) activity in rats in the sham (S), I/R, intralipid (I) and EIso groups (EIso 1, 2 or 4 ml/kg). Serum Cr, BUN, CYC, TNF-α, IL-6, IL-10 concentrations and renal tissue MDA content were significantly increased, and renal tissue SOD activity was markedly decreased after kidneys subjected to 45 min ischemia and 3 h reperfusion compared with the sham group. Compared with the I/R, intralipid and 1 ml/kg EIso groups, serum Cr, BUN, CYC, TNF-α, IL-6 levels and renal tissue MDA content were significantly reduced, and serum IL-10 level and renal tissue SOD activity were markedly increased after 2 or 4 ml/kg EIso pretreatment. Data represent mean ± SD. ^*^P < 0.05 compared to S, ^†^P < 0.05 compared to I/R, ^‡^P < 0.05 compared to I, ^§^P < 0.05 compared to EIso 1 ml/kg.

### Markers of inflammation

Kidneys subjected to 45 min ischemia and 3 h reperfusion significantly increased serum TNF-α, IL-6 and IL-10 concentrations as compared to the sham group (P < 0.05) (Table 2). Both 2 and 4 ml/kg EIso pretreatment markedly reduced serum TNF-α and IL-6 levels, but increased serum IL-10 level, compared with the I/R, intralipid and 1 ml/kg EIso groups (P < 0.05) (Table 2). No significant differences were observed in the three parameters between 2 and 4 ml/kg EIso groups (P > 0.05). There were no significant differences in the three parameters among the I/R, intralipid and 1 ml/kg EIso groups (P > 0.05).

### Markers of oxidative stress

Compared with the sham group, renal tissue MDA content was increased, and SOD activity was decreased after kidneys were subjected to 45 min ischemia and 3 h reperfusion (P < 0.05) (Table 2). Compared with the I/R, intralipid and 1 ml/kg EIso groups, both 2 and 4 ml/kg EIso pretreatment markedly reduced renal tissue MDA content, but increased SOD activity (P < 0.05) (Table 2). There were no significant differences in MDA content and SOD activity between the 2 and 4 ml/kg EIso groups (P > 0.05). No differences were observed in the two parameters among the I/R, intralipid and 1 ml/kg EIso groups (P > 0.05).

### Renal histopathology

**Figure 1:**
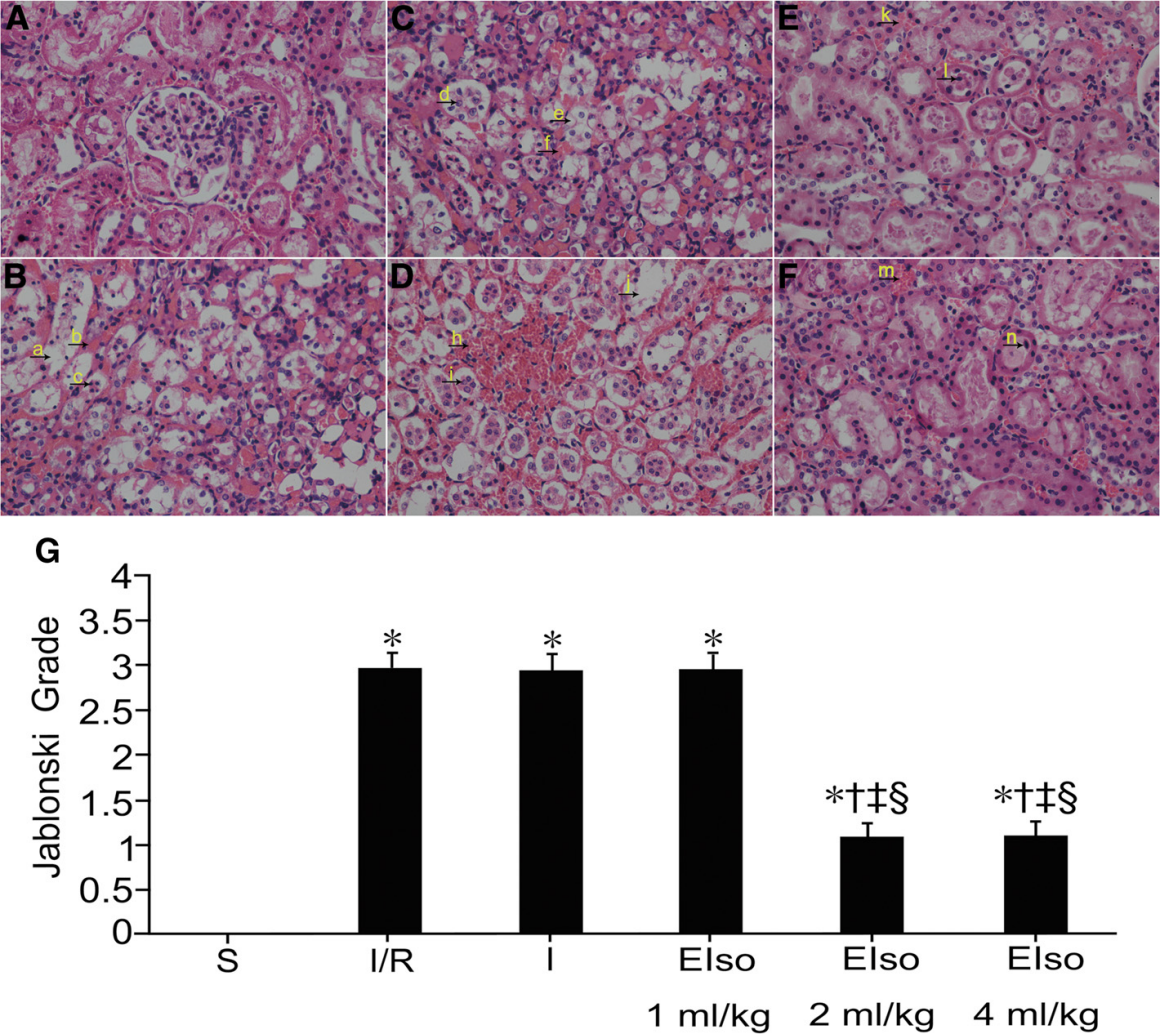
**Renal injury panels (A) to (F) are representative micrographs of optical microscopic analysis in the renal cortex of rats for the sham (S), I/R, intralipid (I) and EIso groups (EIso 1, 2 or 4 ml/kg), respectively.** Normal kidney tissue, normal histological characteristic of glomeruli and tubules were observed in the sham group **(A)**. In the I/R **(B)**, intralipid **(C)** and 1 ml/kg EIso **(D)** groups, marked structural changes were seen in kidney tissues. Arrow a and j indicate tubular dilatation; Arrow b, f and h indicate interstitial hemorrhage; Arrow c, d and i indicate sloughing tubular epithelial cell necrosis; Arrow e indicates tubular epithelial cell vacuolar degeneration. Moderate kidney damages were showed in the 2 **(E)** and 4 **(F)** ml/kg EIso groups. Arrow k and m indicate interstitial hemorrhage; Arrow l and n indicate sloughing tubular epithelial cell necrosis. Original magnification × 400. Panel **(G)** represents Jablonski grade of histological appearance of acute tubular necrosis. The type and severity of acute tubular necrosis was graded from 0 to 4 in each group of rats (see Table 1). Kidneys subjected to 45 min ischemia and 3 h reperfusion increased Jablonski grade as compared to the sham group. Both 2 and 4 ml/kg EIso pretreatment reduced Jablonski grade as compared to the I/R, intralipid and 1 ml/kg EIso groups. ^*^P < 0.05 compared to S, ^†^P < 0.05 compared to I/R, ^‡^P < 0.05 compared to I, ^§^P < 0.05 compared to EIso 1 ml/kg.

The degree of renal necrotic injury is most severe in the proximal tubules located in the renal cortex area after I/R injury. Therefore, we show this area in the figures. Normal histological characteristic of glomeruli and tubules, and no morphological damage was observed in the kidneys of sham group (Figure 1A). Compared with kidneys from the sham group, the kidneys in the I/R, intralipid and 1 ml/kg EIso groups showed significant renal damage demonstrated by severe tubular dilatation, tubular epithelial cell swelling, vacuolar degeneration and necrosis, many tubular epithelial cells sloughing from the basement membrane, tubular luminal obstruction, and interstitial hemorrhage and edema (Figure 1B-D). Rats pretreated with 2 or 4 ml/kg EIso showed marked reduction of the histological features of kidney injury, consisting of focal tubular necrosis and moderate interstitial hemorrhage and edema (Figure 1E-F).

Compared to the sham group (0 ± 0), the histological scores in the other five groups were higher (P < 0.05) (Figure 1G). There were lower histological scores in the 2 and 4 ml/kg EIso groups (1.1 ± 0.7, 1.1 ± 0.6, respectively) compared with the I/R (3.0 ± 0.5), intralipid (2.9 ± 0.5) and 1 ml/kg EIso (3.0 ± 0.4) groups (P < 0.05) (Figure 1G). No differences were observed about the histological scores between the 2 and 4 ml/kg EIso groups (P > 0.05). There were no differences in histological scores among the I/R, intralipid and 1 ml/kg EIso groups (P > 0.05).

## Discussion

In the present study, we demonstrated that 2 ml/kg EIso (8% vol./vol.) pretreatment alleviated renal tissue morphological damage, decreased serum Cr, BUN, CYC, TNF-α and IL-6 concentrations, reduced renal tissue MDA content, increased serum IL-10 concentration and tissue SOD activity in the I/R model of rat kidneys, as 4 ml/kg EIso pretreatment. Our results indicated that EIso may exert protective effects against renal I/R injury in rats by inhibiting systemic inflammatory response and improving antioxidation of the tissue. To our knowledge, this is the first study to show that intravenous administration of EIso could produce renal protection.

Chiari et al. [23] found that intravenous infusion of 6.9% EIso at a constant rate of 3.5 ml/kg per h for 30 min produces acute and delayed preconditioning against myocardial infarction in rabbits. In the same way, intravenous pretreatment of 2 or 4 ml/kg 8% EIso for 30 min affords effective protection against myocardial ischemia in rats [15]. And these doses of EIso had no significant inhibitory effects on circulation function in pentobarbital anesthetized rats [15]. Base on these studies, we demonstrated in the present study that EIso (2 or 4 ml/kg for 30 min) is useful for preventing renal injury after ischemia.

Measurement of serum Cr and BUN levels have been used to determine renal function in many experimental studies. Coll et al. [24] reported that serum concentration of CYC could reflect glomerular filtration rate very accurately, even in cases where there was only a minor reduction in glomerular filtration rate. A meta-analysis of 46 CYC-related studies to evaluate the superiority of CYC levels over serum Cr levels showed that serum CYC is a more potent marker of glomerular filtration rate than serum Cr [25]. Serum CYC level is a more sensitive test for the early detection of renal function impairment or reduced glomerular filtration rate as compared to serum Cr [26]. In the present study, serum Cr, BUN and CYC levels in the 2 and 4 ml/kg EIso groups were markedly decreased compared with the I/R, intralipid and 1 ml/kg EIso groups. In addition, the histological scores of renal injury were significantly decreased with 2 or 4 ml/kg EIso preconditioning. It showed that both 2 and 4 ml/kg EIso pretreatment induce protective effects against renal I/R injury in rats.

With renal I/R injury, necrotic cell death results in further activation of inflammatory cascades, resulting in more severe secondary tissue damage [27]. The inflammatory components consist of TNF-α, IL-6 and IL-8 that cause leukocytes to accumulate in the vasa recta of the outer medulla [28]. There are growing evidences from in vitro and in vivo models that the course of renal I/R injury is associated with intra-renal inflammation, and therefore it is now recognized that intra-renal inflammation is deeply involved in the pathogenesis of renal I/R injury [1]. Increased levels of renal and serum IL-6 were observed in mice subjected to renal I/R [29]. And IL-6-knockout mice suffered less renal I/R injury [30]. The experimental hyperlipidemia induced by poloxamer 407 decreased renal I/R injury, which was mediated by reduced renal IL-6 production after the insult [31]. Lee et al. [9] demonstrated that 1 minimum alveolar concentration isoflurane given during renal I/R protects against renal I/R injury in rats by reducing renal TNF-α concentrations and inhibiting nuclear factor-κB activation. Hashiguchi et al. [10] also showed that 1.5% isoflurane has a preconditioning effect against rat renal I/R injury when administered 20 min before ischemia, which may be related to inhibition of the protein kinases, Jun N-terminal kinase and extracellular signal-regulated kinase. Recently, pretreatment with 1.5% isoflurane was showed to ameliorate renal I/R injury in mice via upregulation of hypoxia inducible factor-1 α [12]. In addition, several studies implicated that isoflurane activates intestinal sphingosine kinase to protect against renal I/R-induced [8] or bilateral nephrectomy-induced [32] liver and intestine injury. Other studies suggested that volatile anesthetics could protect against I/R injury in the heart [33] and lung [34] via anti-inflammatory effects. IL-10 is expressed and secreted by a variety of cell types, which prevents production of proinflammatory cytokines and chemokines by monocytes/macrophages. IL-10 is an antiinflammatory cytokine which contributes to improvement in left ventricle functional recovery after myocardial infarction in a cultured cell study [35]. And IL-10-deficient mice revealed increased neutrophil infiltration, infarct size, and myocardial necrosis after acute myocardial infarction [36]. In the present study, we demonstrated that both 2 and 4 ml/kg EIso preconditioning significantly reduced the serum levels of TNF-α and IL-6, increased the serum level of IL-10 as compared to the I/R, intralipid and 1 ml/kg EIso groups. Our results indicated that EIso-induced protection against renal I/R injury was probably related to preventing inflammatory response. In a recent study, Lv et al. [18] showed that EIso preconditioning decreased nuclear factor-κB activity, TNF-α level, myeloperoxidase activity, and intercellular adhesion molecule-1 expression in the lung, which resulted to reduce lung injury induced by hepatic I/R. Both 2 and 4 ml/kg EIso intravenously infused were also found to attenuate myocardial damage by inhibiting apoptosis after ischemia [15].

It is well known that oxygen free radicals contributes to the pathogenesis of I/R injury. MDA is one of the products of free radical chain reaction and lipid peroxidation. The changes in tissue MDA content reflect changes in the quantity of polyunsaturated fatty acid reductions during I/R injury. Consequently, the increase of MDA content indicates a lowering of the membrane fluidity and impairment of the normal membrane structure of the mitochondria. SOD is one of important antioxidase that scavenges oxygen free radicals and protects mitochondria against damage caused by potentially cytotoxic reactions. Usually antioxidative ability of tissue is evaluated by MDA content and SOD activity. Yurdakoc et al. [37] showed that isoflurane could protect against lipid peroxidation of cerebral injury in rats. Recently, a study demonstrated that EIso increased antioxidation in mitochondria to protect against liver and lung injury in the rat hemorrhagic shock model [38]. Similarly, Xu et al. showed that 2 ml/kg EIso pretreatment could ameliorate lipopolysaccharide-induced acute lung injury in rats by decreasing pulmonary MDA level and increasing pulmonary SOD activity [39]. Our present study showed that both 2 and 4 ml/kg EIso preconditioning resulted in a decrease of MDA content and an increase of SOD activity in the kidney tissue. These findings suggested that EIso pretreatment could enhance renal antioxidative ability, which might be one of mechanisms of renal protection of EIso with 2 or 4 ml/kg, partly protected against I/R injury.

In the present study, EIso pretreated the kidney with 2 or 4 ml/kg against I/R injury in rats. However, the lower dose of EIso (1 ml/kg) did not appear to produce protection, which is consistent with a previous report concerning a dose-dependent effect of isoflurane [40]. There were no significant differences between the 2 and 4 ml/kg EIso groups. The similar result was found in the rat myocardial injury model [15]. Whether formulations containing higher concentrations or different rates of infusion could be responsible for greater renal protection deserves further investigation.

EIso is composed of isoflurane and intralipid. As a vehicle, intralipid may influence the renal protection of this anaesthetic, so the effects of intralipid were also investigated in the present study. It was demonstrated that intralipid preconditioning alone did not protect renal injury. There is a conflicting report regarding the renal protective effect of intralipid during acute renal I/R [41]. Several factors can account for these discrepant results, including differences in experimental design, as well as exposure time and drug concentration. In the present study, there were no significant differences in these variables between the intralipid and I/R groups with a same experimental protocol. Therefore, we concluded that the intralipid could be not responsible for the renal protection of EIso.

There are some limitations of this study, including the brief period of observation, and one blood and tissue sampling time point. Moreover, blood concentrations of isoflurane during infusion and before renal pedicles occlusion were not measured, so unequal doses of isoflurane may administer to rats subjected to renal ischemia.

EIso can be administrated intravenously, which makes its clinical application more practical than inhalation of isoflurane. For this reason intravenous EIso may gain more wider acceptance as a treatment option for clinical application.

## Conclusions

In conclusion, the present data demonstrated that intravenous administration of EIso before occlusion of the renal pedicle could afford effective protection against renal I/R, and attenuate renal proximal tubular dilatation, tubular epithelial cells swelling, necrosis and sloughing, alleviate interstitial hemorrhage and edema in rats, which might be mediated by inhibiting systemic inflammatory response and improving antioxidation of the tissue.

## Abbreviations

I/R: Ischemia/reperfusion; EIso: Emulsified isoflurane; TNF: Tumor necrosis factor; Cr: Creatinine; BUN: Blood urea nitrogen; CYC: Cystatin c; IL: Interleukin; ELISA: Enzyme linked immunosorbent assay; MDA: Malondialdehyde; SOD: Superoxide dismutase.

## Competing interests

The authors declare that they have no competing interests.

## Authors’ contributions

ZQ, JJ and MZ participated in the design of the study, performance of the study, interpretation of results and writing. EL, LZ and XX participated in the design of the study, interpretation of results and writing. All authors have read and approved the final manuscript.

## Pre-publication history

The pre-publication history for this paper can be accessed here:

http://www.biomedcentral.com/1471-2253/14/28/prepub
